# Threshold Effects of Maternal Vitamin C in Late Pregnancy on Infant Social Competence: A Nonlinear Dose‐Response Analysis in a Birth Cohort

**DOI:** 10.1002/fsn3.71289

**Published:** 2025-12-30

**Authors:** Cui Li, Ji Jiafen, Ni Juan, Li Rui xiang

**Affiliations:** ^1^ Shandong Second Medical University Affiliated Hospital Weifang Shandong China

**Keywords:** dose–response curve, neurodevelopmental programming, personal‐social ability, threshold effect, vitamin C

## Abstract

This study aims to investigate the nonlinear dose‐effect relationship between late‐pregnancy serum vitamin C concentration and early personal‐social behavioral abilities in offspring among the northern Chinese population. It will also parse the threshold characteristics and clinical significance of this relationship. This prospective mother‐infant cohort study enrolled 442 mother‐offspring pairs. Late‐pregnancy (28–40 weeks) serum concentrations of vitamins A, E, and C were measured using high‐performance liquid chromatography (HPLC). Offspring social ability at 6 months of age was assessed using the Gesell Developmental Diagnosis Scale combined with the Infant‐Junior High School Student Social Life Ability Scale (S‐M). A generalized additive mixed model (GAMM) and a piecewise linear regression model were constructed to analyze the associations. These analyses were adjusted for confounding factors, including parental demographic characteristics, pregnancy complications, and nutritional supplementation patterns. Univariate analysis revealed significant positive correlations between late‐pregnancy serum vitamin C, A, and E concentrations and offspring social ability (*β* = 0.082, 0.387, 0.039; all *p* < 0.05). Factors associated with improved social ability scores included at least 8 h of daily sleep during pregnancy (*β* = 0.504), an Apgar score of 9 (*β* = 7.825), and intermittent folic acid supplementation (*β* = 1.693) (all *p* < 0.05). Conversely, pregnancy complications (*β* = −1.130) and poor sleep quality (*β* = −1.582) significantly reduced scores (all *p* < 0.05). GAMM and piecewise linear regression analyses demonstrated a significant nonlinear association between late‐pregnancy serum vitamin C concentration and early offspring social ability: the strongest effect occurred at low concentrations (≤ 4.9 μmol/L, *β* = 0.654/μmol, *p* = 0.018); a still‐significant but gradually attenuated positive association was observed at moderate concentrations (4.9–49 μmol/L, *β* = 0.086/μmol, *p* = 0.001); and the effect diminished and became statistically insignificant at high concentrations (≥ 49 μmol/L, *β* = 0.061, *p* = 0.118). The present study discloses a nonlinear dose–response relationship between maternal vitamin C levels in late pregnancy and early offspring social ability. The association was most pronounced at concentrations ≤ 4.9 μmol/L, remained significant but attenuated within the 4.9–49 μmol/L range, and became nonsignificant beyond 49 μmol/L. The optimal window for neurodevelopmental benefit was identified as 23–49 μmol/L. These findings call into question the conventional approach to deficiency supplementation and support the implementation of personalized, biomarker‐guided nutrition strategies during pregnancy to optimize offspring neurodevelopmental outcomes.

## Graphical abstract

This prospective cohort study analysing 442 mother‐offspring pairs reveals a nonlinear dose‐response relationship between late‐pregnancy serum vitamin C concentration and early offspring social competence. The strongest association was observed at low concentrations (≤ 4.9 μmol/L, *β* = 0.654/μmol), with diminishing effects at moderate levels (4.9–49 μmol/L) and no significance at high concentrations (≥ 49 μmol/L). The findings advocate for personalized prenatal vitamin C supplementation strategies based on maternal blood concentration monitoring to optimize neurodevelopmental outcomes.

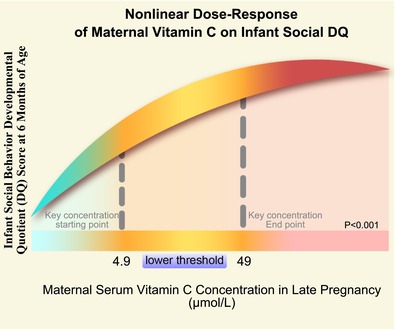

## 1 Introduction

The first 1000 days of life, from pregnancy to 2 years of age, represent a critical “window of opportunity” for children's neurodevelopment. During this period, nutritional status profoundly influences the lifelong structural and functional development of the offspring brain through epigenetic programming and redox homeostasis regulation (Cusick and Georgieff 2016; Barker et al. 2013; Hansen et al. 2018; Cheng 2016; Paidi et al. 2014). Notably, maternal nutrient deficiency may disrupt key processes, such as synaptogenesis and neuronal migration, in the fetal hippocampus and prefrontal cortex, leading to abnormal social behaviors in later life (Paidi et al. 2014; Tveden‐Nyborg et al. 2012; Zhang 2020; Xu et al. 2024). Personal‐social competence, a core dimension of early childhood development, includes complex functions such as imitative behaviors, emotional interaction, and environmental adaptation. Its developmental trajectory is regulated by multiple factors. Genetic susceptibility, family interaction patterns, and prenatal nutritional exposure may all shape “neuroprogramming effects” via epigenetic mechanisms (Plevin and Galletly 2020).

Vitamin C has become a central topic in research because of its diverse neuroprotective effects. It acts as a potent antioxidant and plays multiple roles in neural development (Li et al. 2016). Additionally, acting as a ligand for the SVCT2 transporter, vitamin C activates the JAK2/STAT2 signaling pathway to regulate neural progenitor cell differentiation and synaptogenesis, directly influencing synaptic plasticity and dopaminergic system development (Salazar et al. 2023; Ward et al. 2013). Animal studies confirm that prenatal vitamin C deficiency reduces offspring hippocampal volume by 30%. This reduction is associated with social memory deficits, motor impairments, enhanced dopamine sensitivity, and striatal dysfunction. These effects persist into adulthood. Notably, moderate vitamin C supplementation reverses these impairments (Chen et al. 2012). Population‐based evidence further indicates that each 1 μg/mL increase in maternal vitamin C levels during the second trimester correlates with a 27.2 g rise in birth weight and a 0.17 cm gain in body length (Lee et al. 2004; Chen et al. 2009). Additionally, levels of antioxidant vitamins A, C, and E in umbilical cord blood are positively associated with motor and language abilities in 2‐year‐old children (Chen et al. 2009; Zhang et al. 2009). However, existing research has several limitations. First, most data are derived from Western populations, neglecting how China's diverse dietary patterns affect vitamin C metabolism (Cheng 2016; Coker et al. 2022). Second, prior studies mainly focus on linear associations between vitamin C and composite cognitive scores, with limited investigation into its specific role in social behaviors such as eye contact and joint attention (Huang et al. 2021). Finally, although animal models suggest concentration‐dependent neuroprotection (Zhang et al. 2018), the “optimal intervention window” remains unvalidated in mother‐infant cohorts (Tveden‐Nyborg et al. 2009). Moreover, high‐dose vitamin C may counteract its benefits through pro‐oxidant effects (Li et al. 2016), highlighting the need to establish safe dosage boundaries for nutritional interventions.

This study, conducted in a prospective cohort of healthy mother‐infant pairs in northern China, aims to investigate the dose–response relationship between maternal serum vitamin C levels in late pregnancy and offspring personal‐social competence at 6 months of age. Findings will provide empirical support for optimizing maternal vitamin C monitoring thresholds during pregnancy, thereby contributing to the development of precision nutritional intervention strategies.

## 2 Methods

### 2.1 Study Population

**FIGURE 1:**
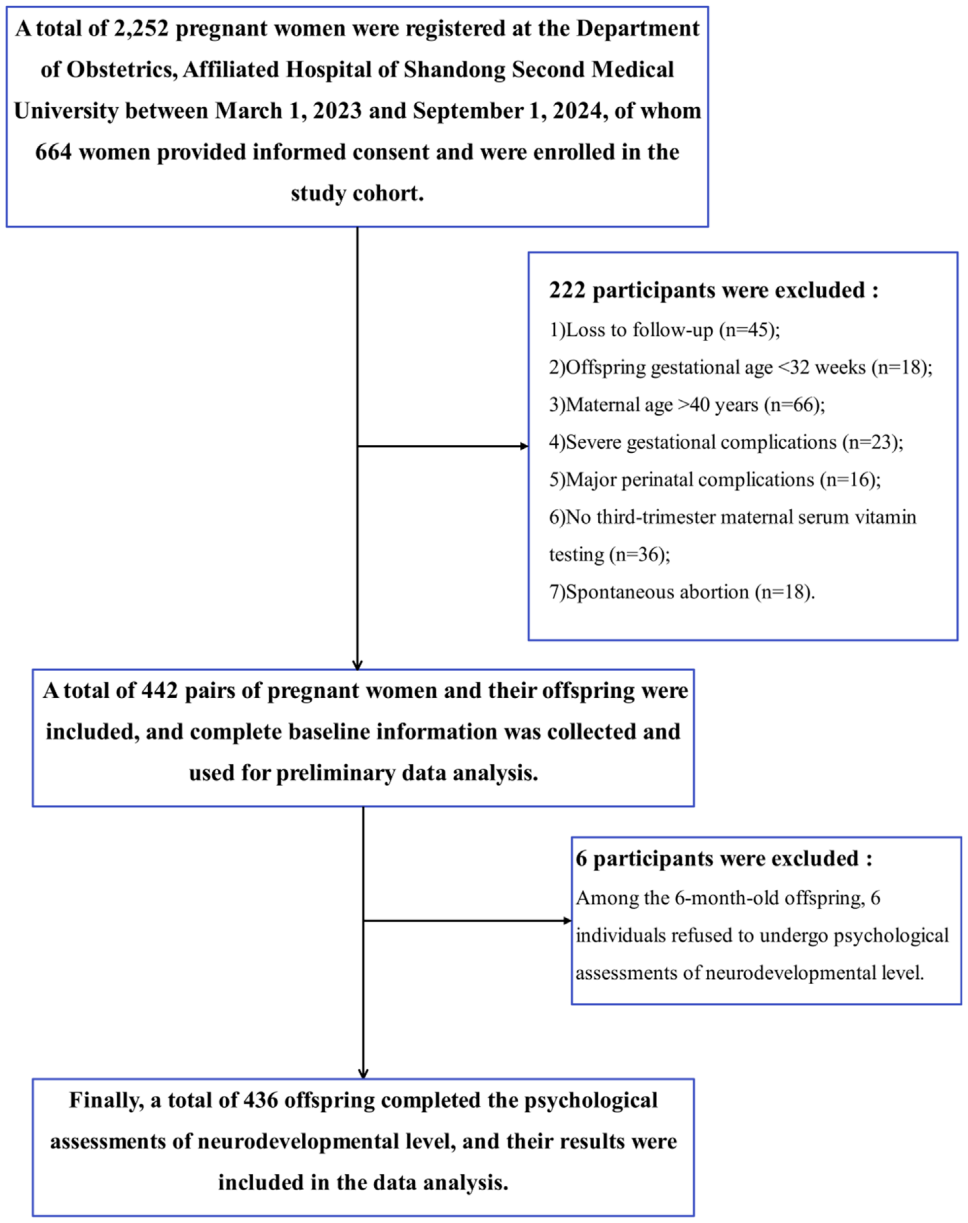
Flowchart of study participant enrollment.

This study selected all pregnant women and their offspring who established medical records at the Obstetrics Department of Shandong Second Medical University Affiliated Hospital from March 1, 2023, to September 1, 2024, and voluntarily participated in this research, thereby forming a mother‐infant birth cohort. Follow‐up commenced from the establishment of medical records until the offspring reached 6 months of age, resulting in a final analysis of 442 mother‐infant pairs (Figure 1). Exclusion criteria included: (1) pregnant women who were either too young (< 14 years) or too old (> 40 years); (2) pregnant women with severe underlying conditions (such as heart disease, neuropsychiatric disorders, hereditary metabolic diseases, or severe connective tissue diseases) or experiencing severe complications during pregnancy (miscarriage, severe preeclampsia, significant fetal growth restriction, severe intrauterine distress, placental abruption, severe cholestasis, etc.); (3) offspring born before 32 weeks of gestation; (4) offspring with diseases that significantly impact growth and brain development (severe hypoxic–ischemic encephalopathy, complex congenital heart disease, severe intracranial hemorrhage, hereditary metabolic disorders, etc.). The research protocol received approval from the Ethics Committee of Shandong Second Medical University Affiliated Hospital (wyfy‐2022‐ky‐260). All participants in this study voluntarily provided informed written consent.

### 2.2 Data Collection

After obtaining informed consent from the pregnant women, a self‐designed questionnaire was employed for face‐to‐face interviews to gather information on the social and demographic characteristics of the pregnant women, as well as factors potentially affecting the growth and development of their offspring. Blood samples (5 mL) were collected from the pregnant women in the late pregnancy stage (28 to 40 weeks) using disposable inert red vacuum collection tubes. All samples were immediately refrigerated and sent in batches to the hospital's laboratory testing center, where they were centrifuged using a high‐speed centrifuge to separate the serum. The concentrations of serum vitamin A, vitamin E, and vitamin C were determined using high‐performance liquid chromatography (HPLC) (Shimadzu Corporation, Japan), following the specific detection methods described by Shamsipur et al. (2018). The physical indicators of the offspring, including birth weight and birth length, were measured by midwives using standard physical measurement techniques. Measurements of height and weight were conducted using a newborn height and weight measuring device (Kangwa WS‐RTG‐1GD (pro), China), with length precise to 0.1 cm and weight to 0.01 kg. Head circumference was measured using a non‐stretchable soft tape, accurate to 0.1 cm. Standard categories for vitamin C status: Deficiency: < 11 μmol/L Insufficiency: 11–23 μmol/L; Adequate: > 23 μmol/L.

### 2.3 Psychometric Assessment of Child Developmental Level

At the 6‐month follow‐up, the neurodevelopmental level of the offspring was assessed using the Gesell Developmental Schedules (GDS), a standardized psychometric tool widely used in China for children aged 0–6 years. The GDS evaluates five key domains: adaptive behavior, gross motor behavior, fine motor behavior, language behavior, and personal‐social behavior. The personal‐social behavior domain specifically measures social competencies, including the infant's response to social cues, interaction with caregivers, and adaptive social behaviors, which align with the study's focus on social development. To ensure reliability and address developmental variability, all assessments were conducted by trained examiners using standardized stimuli (e.g., social gaze elicitation with neutral‐face protocols). Scores were adjusted for gestational age at assessment to account for prematurity‐related variations. the Infant‐Junior High School Student Social Life Ability Scale (S‐M Scale) suitable for infants from 6 months to 15 years, was scored based on item pass rates, with standard scores used for interpretation. This dual‐tool approach‐capturing neurodevelopmental maturation (GDS) and contextually embedded social functioning (S‐M)‐aligns with multidimensional frameworks validated in Chinese cohorts.

Before the assessment, pediatric physicians instructed parents to ensure the child had adequate rest and avoided stressors, minimizing distress or noncompliance during testing. Assessments were conducted by certified assessors trained in standardized protocols (Gesell Institute Certification No. GIC‐2023; S‐M Scale Certification by Peking University First Hospital) in controlled environments (ambient noise < 45 dB, 500–700 lx illumination) adhering to WHO infant testing guidelines. Inter‐rater reliability was ensured through video‐recorded dual scoring (ICC > 0.85).

### 2.4 Statistical Analysis

Data analysis was conducted using R version 4.3.1 (http://www.R‐project.org) and EmpowerStats software (v8.2.4, X&Y Solutions, Boston, MA, USA). Continuous variables were summarized as means ± standard deviations (SDs), while categorical variables were expressed as percentages. For association analysis, simple linear regression estimated relationships between maternal and neonatal factors (e.g., maternal vitamin C intake, neonatal birth weight) and offspring personal‐social behavior DQ. To explore nonlinear effects, a generalized additive mixed model (GAMM) was applied to assess the relationship between maternal serum vitamin C concentration during late pregnancy and offspring personal‐social behavior DQ. Additionally, piecewise linear regression with smoothing functions identified optimal vitamin C concentration thresholds. The breakpoint was selected via trial‐and‐error optimization to maximize model likelihood, with the log‐likelihood ratio test comparing linear vs. piecewise models (α = 0.05, two‐tailed). Effect sizes were reported as *β* coefficients with 95% confidence intervals (CI).

## 3 Results

### 3.1 Baseline Characteristics of the Mother–Infant Pairs

This study enrolled 442 mother‐infant pairs. Maternal serum concentrations of vitamin C, A, and E in late pregnancy were measured as 32.89 ± 20.64, 1.561 ± 0.664, and 20.66 ± 13.09 μmol/L, respectively. Vitamin C status demonstrated a generally favorable profile: 77.60% of mothers (343 cases) had adequate levels (> 23 μmol/L), 5.43% (24 cases) were insufficient (11–23 μmol/L), and 16.97% (75 cases) were deficient (< 11 μmol/L). The mean maternal age was 31.11 ± 4.56 years, and the mean neonatal birth weight was 3244.86 ± 510.22 g. The preterm birth rate was 7.24%. At 6 months, infant developmental quotients (DQ) showed lower scores in adaptability (93.7 ± 5.13) and language ability (93.32 ± 5.24) than in personal‐social competence (97.85 ± 5.02). Additionally, 52.0% of mothers experienced pregnancy complications, 70.4% reported irregular vitamin D supplementation, and 73.5% had intermittent folic acid supplementation (Table 1).

**TABLE 1:** Baseline characteristics of the study population (*n* = 442).

Variable	Mean + SD/*n* (%)
Maternal serum vitamin C (μmol/L)	32.91 ± 20.62
Maternal serum vitamin E (μmol/L)	20.66 ± 13.09
Maternal serum vitamin A (μmol/L)	1.56 ± 0.66
Birth head circumference (cm)	34.84 ± 1.39
Birth length (cm)	49.53 ± 2.05
Birth weight (g)	3244.86 ± 510.21
Ponderal index (kg/m^3^)	2.61 ± 0.23
Head circumference at 6 months (cm)	42.02 ± 1.32
Weight at 6 months (kg)	7.64 ± 0.704
Length at 6 months (cm)	66.834 ± 2.05
Length‐for‐age *z*‐score (LAZ) at 6 months	−0.42 ± 0.80
Weight‐for‐age *z*‐score (WAZ) at 6 months	−0.48 ± 0.78
Gross motor DQ at 6 months (s)	97.35 ± 6.56
Fine motor DQ at 6 months (s)	95.76 ± 6.37
Language and speech DQ at 6 months (s)	93.32 ± 5.24
Personal‐social DQ at 6 months (s)	97.85 ± 5.02
Adaptive behavior DQ at 6 months (s)	93.71 ± 5.13
Social living ability score at 6 months	9.15 ± 0.64
Pre‐pregnancy weight (kg)	62.77 ± 10.50
Maternal height (cm)	163.37 ± 5.10
Maternal age (years)	31.11 ± 4.56
Maternal Sleep Duration During Pregnancy (h/day)	8.46 ± 0.93
Paternal weight (kg)	78.72 ± 14.12
Paternal height (cm)	175.15 ± 8.07
*Infant sex*	
Female	167 (37.78)
Male	275 (62.22)
*Gestational age*	
Term	410 (92.76)
Preterm	32 (7.24)
*Standard categories for vitamin C status*	
≤ 4.9 μmol/L	41 (9.28)
4.9–11 μmol/L	34 (7.69)
11–23 μmol/L	22 (4.98)
23–49 μmol/L	280 (63.35)
49 μmol/L	65 (14.71)
*Apgar score at 1 min of birth (s)*	
8	5 (1.13)
9	8 (1.81)
10	429 (97.06)
*Umbilical cord condition*	
Normal	292 (66.06)
Abnormal (single umbilical artery, spiral cord, short cord, etc.)	150 (33.94)
*Age of complementary food introduction*	
4.5 months	45 (10.181)
5 months	170 (38.46)
After 6 months	227 (51.36)
*Feeding method*	
Breastfeeding	229 (51.81)
Mixed feeding	105 (23.76)
Formula feeding	108 (24.43)
*Placental condition*	
Normal	434 (98.19)
Abnormal	8 (1.81)
*Pregnancy sleep quality*	
Good	357 (80.77)
Poor	85 (19.23)
*Frequency of nighttime awakenings during pregnancy*	
None	74 (16.74)
Occasionally	344 (77.83)
Frequently	24 (5.43)
*Vitamin D supplementation during pregnancy*	
None	56 (12.67)
Occasionally	311 (70.36)
As prescribed by doctor	75 (16.97)
*Vitamin E supplementation during pregnancy*	
None	194 (43.89)
Occasionally	182 (41.18)
As prescribed by doctor	66 (14.93)
*Folic acid supplementation*	
As prescribed by doctor	92 (20.81)
Intermittent	325 (73.53)
None	25 (5.66)
*Anemia during pregnancy*	
Yes	131 (29.64)
No	311 (70.36)
*Pregnancy comorbidities (hypertension, diabetes, etc.)*	
Yes	230 (52.04)
No	212 (47.96)
*Maternal pre‐pregnancy health status*	
Yes (pre‐existing conditions)	54 (12.22)
No	388 (87.78)
*Maternal income (CNY/month)*	
< 3000	102 (23.08)
3000–6000	322 (72.85)
6000–10,000	18 (4.072)
*Maternal grandparents' health status*	
Poor	128 (28.959)
Good	314 (71.041)
*Maternal occupation*	
Government/enterprise cadre	8 (1.81)
Professional/technical	83 (18.78)
Worker	10 (2.26)
Farmer (forestry, animal husbandry, fishery)	6 (1.36)
Commercial/service	35 (7.92)
Medical staff	11 (2.49)
Other	289 (65.39)
*Maternal education level*	
Primary school or below	3 (0.68)
Junior high school	16 (3.62)
Senior high school	218 (49.32)
College/university	197 (44.57)
Graduate school	8 (1.81)
*Father's family health status*	
Affected by chronic diseases (e.g., hypertension, diabetes)	4 (0.91)
In good health	438 (99.10)
*Father's occupation*	
Government/institutional cadres	16 (3.62)
Professional and technical personnel	42 (9.50)
Workers	43 (9.73)
Farmers (forestry, animal husbandry, fishery)	8 (1.81)
Commerce and service workers	28 (6.33)
Other	305 (69.01)
*Father's education level*	
Junior high school	14 (3.17)
Senior high school	204 (46.15)
College/university	218 (49.32)
Graduate school	6 (1.36)

* Continuous variables are presented as mean ± standard deviation (SD); categorical variables are presented as frequency (percentage).

### 3.2 Influencing Factors of Offspring's Personal‐Social Competence at 6 Months of Age

Univariate analysis (Table 2) showed significant positive correlations between offspring's personal‐social competence at 6 months and maternal serum vitamin levels in late pregnancy. Specifically, vitamin C (*β* = 0.082, 95% CI [0.061–0.104], *p* < 0.001), vitamin A (*β* = 0.387, 95% CI [0.134–0.640], *p* = 0.003), and vitamin E (*β* = 0.039, 95% CI [0.002–0.075], *p* = 0.042) were all positively associated. Maternal behaviors and prenatal factors also showed robust associations: sufficient sleep duration (≥ 8 h/day, *β* = 0.504, 95% CI [0.002–1.006], *p* = 0.049), a 1‐min Apgar score of 9 (vs. 8, *β* = 7.825, 95% CI [2.267–13.383], *p* = 0.006), and intermittent folic acid supplementation (vs. prescribed, *β* = 1.693, 95% CI [0.538–2.848], *p* = 0.004).

**TABLE 2:** Univariate analysis of factors associated with individual‐social behavior in 6‐month‐old offspring (*n* = 442).

Variable	Statistic	*β* (95% CI)	*p*
Maternal serum vitamin C (μmol/L)	32.91 ± 20.62	0.082 (0.06, 0.10)	***< 0.001***
Maternal serum vitamin E (μmol/L)	20.66 ± 13.09	0.387 (0.13, 0.64)	***0.002***
Maternal serum vitamin A (μmol/L)	1.56 ± 0.66	0.039 (0.002, 0.07)	***0.03***
Birth head circumference (cm)	34.84 ± 1.39	0.014 (−0.33, 0.36)	0.93
Birth length (cm)	49.53 ± 2.05	−0.170 (−0.39, 0.06)	0.14
Birth weight (g)	3244.86 ± 510.21	−0.000 (−0.001, 0.001)	0.85
Ponderal index (kg/m^3^)	2.61 ± 0.23	1.028 (−0.88, 2.93)	0.29
Pre‐pregnancy weight (kg)	62.77 ± 10.50	−0.012 (−0.06, 0.03)	0.61
Maternal height (cm)	163.37 ± 5.10	−0.065 (−0.16, 0.03)	0.17
Maternal age (years)	31.11 ± 4.56	−0.011 (−0.12, 0.09)	0.84
Maternal Sleep Duration During Pregnancy (hours/day)	8.46 ± 0.93	0.504 (0.002, 1.012)	***0.04***
Paternal weight (kg)	78.72 ± 14.12	−0.028 (−0.06, 0.005)	0.09
Paternal height (cm)	175.15 ± 8.07	0.003 (−0.06, 0.06)	0.92
Head circumference at 6 months (cm)	42.02 ± 1.32	0.002 (−0.36, 0.36)	0.99
Weight at 6 months (kg)	7.64 ± 0.704	−0.539 (−1.22, 0.14)	0.12
Length at 6 months (cm)	66.834 ± 2.05	−0.017 (−0.25, 0.22)	0.89
*Age of complementary food introduction*			
4.5 months	45 (10.181)	Ref	
5 months	170 (38.46)	−0.499 (−2.160, 1.163)	0.46
After 6 months	227 (51.36)	0.561 (−1.055, 2.177)	0.49
*Feeding method*			
Breastfeeding	229 (51.81)	Ref	
Mixed feeding	105 (23.76)	−1.271 (−2.44, −0.11)	0.03
Formula feeding	108 (24.43)	−0.296 (−1.44, 0.85)	0.61
*Apgar score at 1 min of birth (s)*			
8	5 (1.13)	Ref	
9	8 (1.81)	7.825 (2.27, 13.38)	***0.006***
10	429 (97.06)	2.994 (−1.39, 7.38)	0.18
*Umbilical cord condition*			
Normal	292 (66.06)	Ref	
Abnormal (single umbilical artery, spiral cord, short cord, etc.)	150 (33.94)	−0.466 (−1.461, 0.528)	0.36
*Infant sex*			
Female	167 (37.78)	Ref	
Male	275 (62.22)	0.451 (−0.52, 1.42)	0.36
*Gestational age*			
Term	410 (92.76)	Ref	
Preterm	32 (7.24)	2.254 (0.46, 4.05)	***0.01***
*Pregnancy sleep quality*			
Good	357 (80.77)	Ref	
Poor	85 (19.23)	−1.582 (−2.77, −0.39)	***0.01***
*Frequency of nighttime awakenings during pregnancy*			
None	74 (16.74)	Ref	
Occasionally	344 (77.83)	−0.299 (−1.56, 0.96)	0.64
Frequently	24 (5.43)	0.973 (−1.33, 3.28)	0.41
*Vitamin D supplementation during pregnancy*			
None	56 (12.67)	Ref	
Occasionally	311 (70.36)	0.685 (−0.75, 2.12)	0.35
As prescribed by doctor	75 (16.97)	0.488 (−1.25, 2.23)	0.58
*Vitamin E supplementation during pregnancy*			
None	194 (43.89)	Ref	
Occasionally	182 (41.18)	0.774 (−0.25, 1.80)	0.14
As prescribed by doctor	66 (14.93)	−0.166 (−1.57, 1.24)	0.82
*Folic acid supplementation*			
As prescribed by doctor	92 (20.81)	Ref	
Intermittent	325 (73.53)	1.693 (0.54, 2.85)	***0.004***
None	25 (5.66)	1.347 (−0.85, 3.55)	0.23
*Anemia during pregnancy*			
Yes	131 (29.64)	Ref	
No	311 (70.36)	0.225 (−0.81, 1.26)	0.67
*Pregnancy comorbidities (hypertension, diabetes, etc.)*			
Yes	230 (52.04)	Ref	
No	212 (47.96)	−1.130 (−2.07, −0.19)	***0.02***
*Maternal pre‐pregnancy health status*			
Yes (pre‐existing conditions)	54 (12.22)	Ref	
No	388 (87.78)	1.243 (−0.18, 2.67)	0.09
*Maternal income (CNY/month)*			
< 3000	102 (23.08)	Ref	
3000–6000	322 (72.85)	−0.306 (−1.43, 0.82)	0.60
6000–10,000	18 (4.072)	−1.408 (−3.93, 1.11)	0.27
*Maternal grandparents' health status*			
Poor	128 (28.959)	Ref	
Good	314 (71.041)	0.591 (−0.45, 1.63)	0.27
*Maternal occupation*			
Government/enterprise cadre	8 (1.81)	Ref	
Professional/technical	83 (18.78)	1.015 (−2.65, 4.68)	0.59
Worker	10 (2.26)	1.725 (−2.96, 6.41)	0.47
Farmer (forestry, animal husbandry, fishery)	6 (1.36)	2.625 (−2.71, 7.96)	0.33
Commercial/service	35 (7.92)	1.496 (−2.37, 5.37)	***0.45***
Medical staff	11 (2.49)	0.670 (−3.92, 5.26)	0.774
Other	289 (65.39)	1.199 (−2.44, 4.84)	0.52
*Maternal education level*			
Primary school or below	3 (0.68)	Ref	
Junior high school	16 (3.62)	3.937 (−2.24, 10.12)	0.21
Senior high school	218 (49.32)	5.186 (−0.52, 10.89)	0.07
College/university	197 (44.57)	4.655 (−1.06, 10.37)	0.11
Graduate school	8 (1.81)	4.125 (−2.53, 10.78)	0.22
*Father's family health status*			
Poor	4 (0.91)	Ref	
Good	438 (99.10)	0.604 (−4.34, 5.55)	0.81
*Father's occupation*			
Government/institutional cadres	16 (3.62)	Ref	
Professional and technical personnel	42 (9.50)	2.851 (−0.02, 5.72)	0.05
Workers	43 (9.73)	1.456 (−1.41, 4.32)	0.31
Farmers (forestry, animal husbandry, fishery)	8 (1.81)	4.875 (0.64, 9.11)	***0.02***
Commerce and service workers	28 (6.33)	2.518 (−0.55, 5.58)	0.10
Other	305 (69.01)	2.995 (0.49, 5.50)	***0.02***
*Father's education level*			
Junior high school	14 (3.17)	Ref	
Senior high school	204 (46.15)	1.133 (−1.56, 3.83)	0.41
College/university	218 (49.32)	0.645 (−2.05, 3.34)	0.64
Graduate school	6 (1.36)	−4.571 (−9.34, 0.20)	0.06

Abbreviation: DQ, developmental quotient.

* Continuous variables are presented as mean ± standard deviation (SD); Categorical variables are presented as frequency (percentage).

In contrast, adverse maternal conditions showed significant negative impacts, including pregnancy complications (*β* = −1.130, 95% CI [−2.067 to −0.193], *p* = 0.017), poor sleep quality (*β* = −1.582, 95% CI [−2.768 to −0.396], *p* = 0.010), and mixed feeding (vs. exclusive breastfeeding, *β* = −1.271, 95% CI [−2.437 to −0.105], *p* = 0.033). Paternal occupation showed a dose‐dependent protective effect, with farmers (*β* = 4.875, 95% CI [0.642 to 9.108], *p* = 0.024) and other professions (*β* = 2.995, 95% CI [0.480 to 5.504], *p* = 0.019) both associated with higher social competence. Notably, neonatal anthropometric indices (weight, length, head circumference) and parental socioeconomic factors (income, education) were not statistically significant.

### 3.3 Nonlinear Association Between Late‐Pregnancy Serum Vitamin C Levels and Offspring Personal‐Social Competence at 6 Months

**FIGURE 2:**
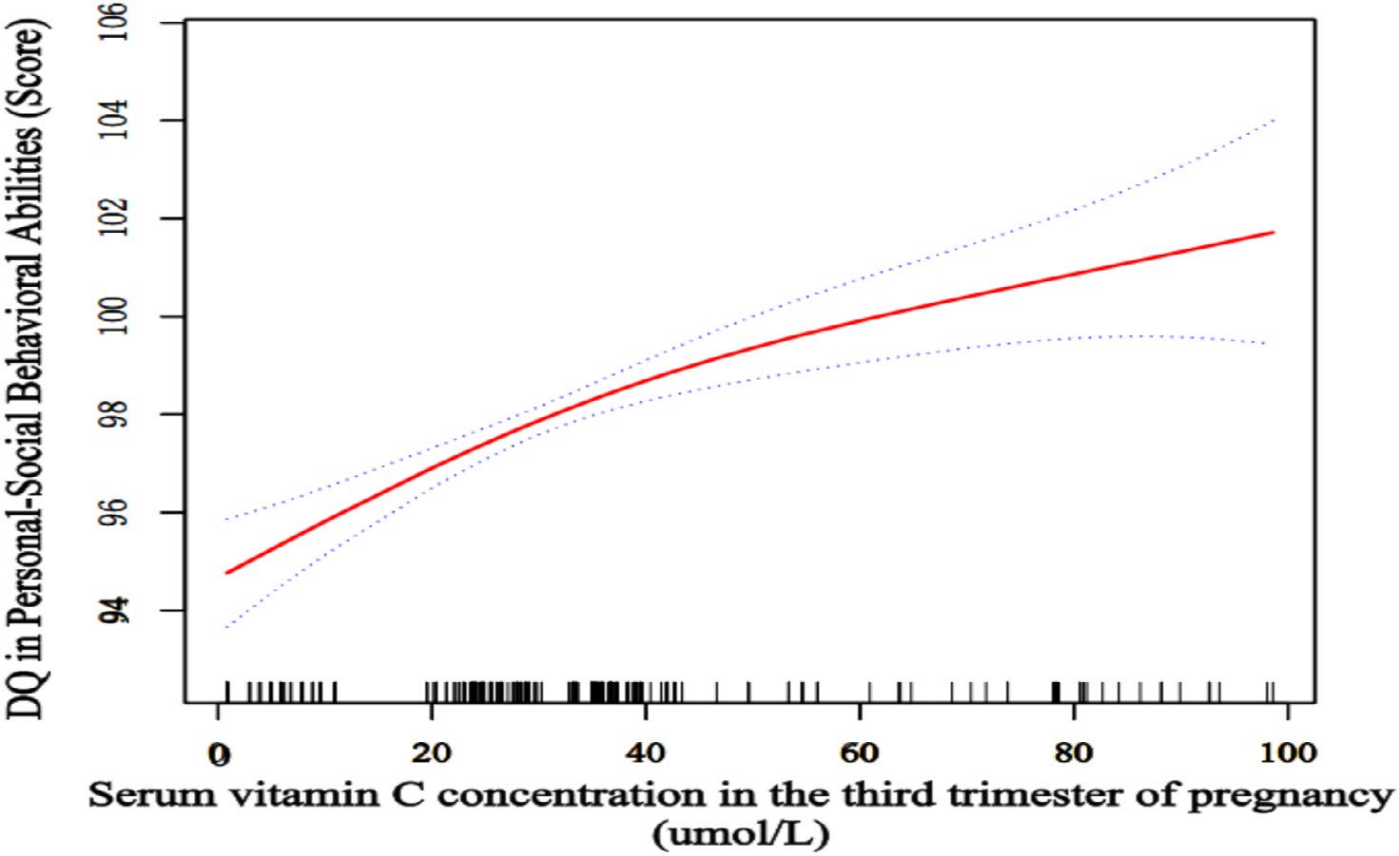
Association between serum vitamin C concentration in the third trimester of pregnancy and Developmental Quotient (DQ) in personal‐social behavioral abilities of 6‐month‐old Infants (*n* = 436)*. *GAMM models were adjusted for offspring parity; Offspring sex; Gestational age; Birth head circumference (cm); Birth length (cm); Birth weight (g); Maternal sleep duration during pregnancy (hours/day); Maternal vitamin E supplementation during pregnancy; Maternal vitamin D3 supplementation during pregnancy; Pregnancy maintenance therapy; Placental status; Maternal grandparents' health status; Paternal grandparents' health status; Paternal height (cm); Paternal weight (kg); Maternal age (years); Maternal income (CNY/month); Maternal residential area; Maternal occupation; Maternal height (cm); Maternal pre‐pregnancy weight (kg); Late‐pregnancy serum vitamin A (μmol/L); Late‐pregnancy serum vitamin E (μmol/L); Prenatal multivitamin/mineral supplementation; Folic acid supplementation (dose/frequency); Maternal sleep duration (hours/day); Exclusive breastfeeding vs. mixed/formula feeding.

To further characterize the dose–response relationship, we employed generalized additive mixed models (GAMM) adjusting for maternal age, parity, gestational age, neonatal anthropometry, and socioeconomic factors. These models revealed a significant nonlinear dose–response relationship between maternal serum vitamin C levels in late pregnancy and offspring Personal‐Social Competence Developmental Quotient (DQ) at6 months (*p* < 0.001) (Figure 2). DQscores showed a steeper upward trajectory with increasing vitamin C concentrations in the deficiency range (< 11 μmol/L), followed by gradually diminishing returns in the moderate range, and a plateau in the high range. This triphasic pattern was consistent across sensitivity analyses using restricted cubic splines.

### 3.4 Threshold Effect of Maternal Serum Vitamin C Concentration During Pregnancy on Offspring's Personal‐Social Behavioral Ability at 6 Months of Age

Based on the nonlinear pattern shown in Figure 2, we performed piecewise linear regression to quantify threshold effects, adjusting for multiple confounding factors. The results (Table 3) showed that during the vitamin C deficiency stage (≤ 4.9 μmol/L), each 1 μmol/L increase in maternal serum vitamin C concentration was associated with a significant 0.65‐point increase in offspring's personal‐social behavioral ability DQ (*p* = 0.018). In the concentration range of 4.9 to 49 μmol/L, each 1 μmol/L increase was associated with a 0.086‐point increase in DQ (*p* = 0.001). When the concentration exceeded 49 μmol/L, the effect of vitamin C on offspring's ability decreased to 0.061 and was not statistically significant (*p* = 0.118). Overall, these findings suggest that appropriate vitamin C supplementation in mothers during late pregnancy positively impacts offspring's social behavioral development.

**TABLE 3:** Threshold effect of third‐trimester maternal serum vitamin C on personal–social behavioral ability at 6 months of age analyzed by piecewise linear regression (*n* = 436).

Variable	Adjusted *β* (95% CI)	*p*	Crude *β* (95% CI)	*p*
*personal‐social behavioral ability DQ (S)*				
Serum vitamin C concentration ≤ 4.9 (μmol/L)	0.654 (0.114, 1.194)	0.018	0.776 (0.246, 1.306)	0.004
Serum vitamin C concentration 4.9–49 (μmol/L)	0.086 (0.035, 0.136)	0.001	0.091 (0.040, 0.142)	0.001
Serum vitamin C concentration ≥ 49 (μmol/L)	0.061 (−0.015, 0.138)	0.118	0.068 (−0.009, 0.145)	0.085

*Note:* Crude model: Adjusted for gender and gestational age. Adjusted model: The adjustment factors demonstrated a nonlinear association mirroring the pattern in Figure 2. Breakpoints were determined by maximizing log‐likelihood ratios, with statistical significance tested using two‐tailed *α* = 0.05.

## 4 Discussion

This study provides the first evidence of a nonlinear dose–response relationship between maternal serum vitamin C concentration in late pregnancy and early personal‐social ability development in offspring within a northern Chinese mother–child cohort. Our analysis using mixed‐effects models demonstrated a concentration threshold effect: when serum concentration was ≤ 4.9 μmol/L, each 1‐unit increase corresponded to the greatest improvement in social ability (*β* = 0.654); between 4.9 and 49 μmol/L, a significant positive correlation remained (*β* = 0.086), although the effect gradually weakened; beyond 49 μmol/L, the effect was no longer statistically significant. These findings suggest a clear upper threshold for the influence of vitamin C on offspring neuropsychological development. Furthermore, maternal vitamin C concentrations in pregnant women from northern China are generally suboptimal. Additionally, the study identified independent effects of vitamin A, vitamin E, and maternal sleep quality during pregnancy on early offspring social ability. However, after adjusting for confounding factors, the dose‐effect of vitamin C remained stable. These findings reveal a clear upper threshold for vitamin C's influence on offspring neuropsychological development, challenging the traditional linear supplementation paradigm.

Notably, our findings both align with and extend previous research on antioxidant vitamins and neurodevelopment (Coker et al. 2022; Tveden‐Nyborg et al. 2009; Yang et al. 2025; Wang et al. 2024; Sharma et al. 2022; Hansen et al. 2014). While a recent animal study by Coker et al. provided important mechanistic insights into the timing and sex‐specific effects of maternal vitamin C intake (Briggs‐Gowan et al. 2004). The research did not assess the effects of vitamin C on offspring social competence or cognitive functions. These are critical dimensions to human early development. The present study makes a substantial contribution to the field by demonstrating, for the first time in a human population, that late‐pregnancy serum vitamin C concentration exhibits a nonlinear dose–response relationship with offspring personal‐social ability at 6 months, thereby identifying an optimal concentration window (4.9–49 μmol/L). The present findings offer significant advances in the field through three fundamental aspects in relation to the work of Coker et al. Firstly, the transition from physiological phenotypes to behavioral outcomes is pivotal. The present study explores the extension of vitamin C's programming effects from growth and metabolic indicators to human social behavior development. Secondly, it examines the transition from “deficiency‐supplementation” to “optimal window.” The present study proposes a novel approach that challenges the conventional linear paradigm by examining the nonlinear dose‐effect curve of vitamin C in human subjects. The third objective is to transition from mechanistic speculation to clinical guidance. The identification of threshold concentrations enables the establishment of direct, actionable clinical reference values for the development of prenatal nutrition monitoring systems.

Compared with Chen et al. who reported that umbilical cord blood vitamin C levels are positively correlated with motor and composite developmental scores (Chen et al. 2009). Our study further demonstrates a beneficial effect of vitamin C on personal‐social ability within a specific concentration range (4.9–49 μmol/L). This suggests that distinct antioxidant vitamins may influence specific dimensions of neurodevelopmental behavior through divergent biological pathways. Although scholars such as Tveden‐Nyborg (Hansen et al. 2014) and Pernille Vogt (Tveden‐Nyborg et al. 2012) have focused on the impairments of vitamin C deficiency on hippocampal structure and spatial memory, they did not explore its relationship with social behavior or identify concentration‐dependent effects. Importantly, the mechanism proposed by Coker (Coker et al. 2022) and Plevin (Plevin and Galletly 2020) proposed a mechanism whereby vitamin C influences neurodevelopment via TET‐mediated DNA demethylation. This mechanism may explain the threshold effect observed here: at low concentrations, vitamin C enhances synaptic plasticity through epigenetic modification; at high concentrations, its antioxidant activity might mask these specific regulatory functions. Additionally, Hansen et al. (Tveden‐Nyborg et al. 2009) systematically reviewed the association between vitamin C deficiency and cognitive impairment but did not address effect variations across normal or high concentration ranges. By characterizing the “dose–response” curve of vitamin C in a mother–child cohort for the first time, our study is the first to characterize the “dose–response” curve of vitamin C in a mother–child cohort. This supplements the traditional “deficiency‐supplementation” paradigm, which previously focused only on nutritional thresholds. Furthermore, it provides novel evidence for optimizing prenatal nutrition interventions.

The nonlinear association we observed is likely regulated through multiple synergistic biological mechanisms. First, vitamin C maintains redox homeostasis in the fetal brain by scavenging reactive oxygen species (ROS) and inhibiting lipid peroxidation. Within the low‐concentration range (≤ 4.9 μmol/L), this effect depends significantly on dose and is mediated by enhanced activity of hippocampal superoxide dismutase (SOD) (Cline et al. 2019; Teixeira et al. 2021). Second, as a critical cofactor of TET dioxygenase, vitamin C exerts epigenetic regulation by modulating DNA demethylation of social behavior‐related genes such as SOCS3 and OXTR in the prefrontal cortex. Its promoting effect on H3K4me3 histone modification peaks at moderate concentrations (4.9–49 μmol/L) (Talpalar et al. 2013). Notably, high vitamin C levels (≥ 49 μmol/L) reduce norepinephrine synthesis by chelating iron and inhibiting dopamine *β*‐hydroxylase activity (Surai and Earle‐Payne 2022; Hongsawong et al. 2021; He et al. 2024). Additionally, vitamin C inhibits matrix metalloproteinase‐9 (MMP‐9) activity to maintain blood–brain barrier integrity (Zhou et al. 2021; Ning et al. 2024; Kim et al. 2022) and reduces pro‐inflammatory cytokine IL‐6 levels, alleviating neuroinflammation associated with gestational hypertension (Biswas et al. 2024; Bhol et al. 2024). While vitamin C enhances iron absorption to optimize myelination (Rao 2024; Liu et al. 2025), excessive intake may induce oxidative damage through the Fenton reaction (Hongsawong et al. 2021; He et al. 2024; Tan et al. 2024), highlighting the critical importance of maintaining dynamic balance between vitamin C and trace elements in neurodevelopment.

Together, these findings establish a three‐part regulatory framework involving antioxidation, epigenetics, and neurotransmitters, which provides mechanistic evidence supporting precision nutritional intervention with vitamin C during pregnancy.

Despite these significant findings, we acknowledge that a single measurement may not fully reflect the dynamic nutritional trajectory throughout pregnancy.

Vitamin C deficiency (< 11 μmol/L) was present in 17% of the cohort, which likely reflects chronic nutritional insufficiency rather than acute late‐pregnancy deficiency. This persistent deficiency may have impaired early embryonic developmental processes (e.g., neural crest cell migration or early neurogenesis) (Coker et al. 2024), thereby establishing a developmental trajectory captured by our late‐pregnancy measurement. To better characterize the optimal range, we subdivided vitamin C status into: severe deficiency (< 11 μmol/L, 16.97%), borderline deficiency (11–23 μmol/L, 5.43%), suboptimal but responsive (23–49 μmol/L, 63.35%), and supra‐optimal range (≥ 49 μmol/L, 14.71%). This refined classification reveals that only 63.35% of mothers fall within the range where the most effective neurodevelopmental benefits per unit concentration increase are observed. This updated grading method aligns closely with the concept proposed by Maxwell et al. (Carr and Lykkesfeldt 2021), suggesting that the biological effects of vitamin C exist along a continuous physiological spectrum. Traditional deficiency‐prevention thresholds (e.g., < 11 μmol/L) may inadequately capture the gradient effects of varying concentrations within the “sufficient” range (> 23 μmol/L) on specific physiological functions (Carr and Lykkesfeldt 2021). A substantial proportion of pregnant women in our cohort (36.65%) had vitamin C concentrations outside the range of optimal neurodevelopmental benefit (below 4.9 μmol/L or above 49 μmol/L), highlighting a significant public health opportunity: shifting from deficiency prevention to functional optimization. Future research should integrate serial measurements (addressing temporal dimensions) with functional gradient concentration banding (addressing dose–response precision) to comprehensively elucidate the critical window and optimal concentration range for vitamin C's neurodevelopmental benefits. Prenatal care could thereby move beyond one‐size‐fits‐all supplementation strategies toward personalized nutrient monitoring and timely interventions.

Regarding the reliability of social competency assessment in 6‐month‐old infants—a period characterized by significant developmental variability, we implemented a rigorous methodological framework to ensure measurement validity. We employed a dual‐tool approach using the Gesell Developmental Schedules (GDS) and the Infant‐Junior High School Student Social Life Ability Scale (S‐M Scale), both of which have demonstrated excellent reliability in Chinese infant populations (Briggs‐Gowan et al. 2004; Chen et al. 2021).

All assessments were conducted by certified examiners using standardized protocols in controlled environments, with raw scores adjusted for gestational age at assessment. Furthermore, in our statistical analysis, we adjusted for key potential confounders including birth weight, corrected gestational age, sex, parental education, and household income using multiple linear regression models. This statistical approach helps isolate the independent association between vitamin C exposure and social competence by reducing the variability introduced by inherent developmental diversity and socioeconomic factors. Therefore, while we acknowledge the inherent challenges in measuring social abilities in early infancy, the combination of psychometrically robust tools, standardized administration, and sophisticated statistical control enhances our confidence that the observed nonlinear associations are valid and not merely artifacts of measurement unreliability or uncontrolled confounding.

In conclusion, this study systematically revealed a nonlinear threshold effect between maternal late‐pregnancy serum vitamin C concentration and offspring social ability, identifying the 4.949 μmol/L range as the optimal intervention window. The present study proposes a novel regulatory framework involving antioxidation, epigenetics, and neurotransmitters, and demonstrates its clinical translational value for precision nutritional interventions. However, it is important to consider several limitations: the single time‐point measurement of vitamin C concentration, geographical sample bias, and some unmeasured confounding factors. It is recommended that future studies concentrate on validating these thresholds through multicentre, transregional cohort studies. In addition, it is advised that long‐term effects be elucidated through lifespan tracking and neuroimaging techniques.

Furthermore, interaction networks between vitamin C and other micronutrients should be explored in order to refine precision models for prenatal nutritional interventions.

The present study proposes the adoption of personalized, biomarker‐guided nutrition strategies with the aim of achieving and maintaining concentrations within an optimal range (e.g., 23–49 μmol/L) throughout pregnancy, with a view to maximizing neurodevelopmental benefits in offspring.

## Author Contributions

**Li Rui xiang:** conceptualization (equal), data curation (equal), methodology (equal), project administration (equal), resources (equal), supervision (equal), writing – review and editing (equal). **Cui Li:** data curation (equal), investigation (equal), software (equal), writing – original draft (equal). **Ji Jiafen:** conceptualization (equal), data curation (equal), funding acquisition (equal), investigation (equal), methodology (equal), software (equal), supervision (equal), writing – review and editing (equal). **Ni Juan:** data curation (equal), formal analysis (equal), investigation (equal), software (equal), writing – review and editing (equal).

## Funding

The Impact of Maternal Antioxidant Vitamin Levels on Offspring's Ponderal Index and Early Life Neurocognitive Development (LH2022GG12) and Effectiveness Study of Functional Medicine Technology in Treating Children with Attention‐Deficit/Hyperactivity Disorder (SDWS2025147).

## Disclosure

*Controlled access protocol*: Qualified researchers may request access to de‐identified datasets through the following process: (1) Submit a formal application to the corresponding author (lirx@sdsmu.edu.cn), including: A scientifically valid research proposal; Institutional Ethics Committee. (2) approval certificate; Signed Data Use Agreement. The Data Access Committee will conduct a dual review within 30 working days, assessing. (1) Alignment with original study objectives. (2) Data security protocols (e.g., ISO 27001 compliance).

## Conflicts of Interest

The authors declare no conflicts of interest.

## Data Availability

The datasets generated and/or analyzed during the current study are not publicly available due to the following restrictions: (1) Ethical Compliance: This study involves sensitive medical records of pregnant women and neonates, which contain personally identifiable information protected under China's Regulations on Human Genetic Resources Management and the Ethical Review Measures for Life Sciences and Medical Research Involving Humans (2023). Full anonymization of these datasets would compromise their scientific validity for longitudinal studies. (2) Institutional Policies: Data sharing is restricted by the Institutional Review Board of Shandong Second Medical University Affiliated Hospital (Approval No. wyfy‐2022‐ky‐260), which mandates custodianship of perinatal health data to prevent unauthorized secondary use.
